# American Medical Association

**Published:** 1851-06

**Authors:** 


### AMERICAN MEDICAL ASSOCIATION.

*Fourth Annual Meeting, held in Charleston, May 6th,* 1851.

The Association met at 11 o'clock, at St. Andrew's Hall, Broad street, the President, Dr. Mussey, in the Chair, and Dr. H. W. Desaussure, Secretary.

The Association having been organized, Dr. Thomas Y. Simons, the Chairman of the Committee of the South Carolina Medical Association, in a warm and hearty address, welcomed the Delegates present from the other States, to the City and State, on behalf of his associates, which was responded to in a becoming manner by the President.

Dr. Frost, on behalf of the Committee of Arrangements, read the list of Delegates present—198 present and 27 States represented.

The President of the Association read a letter from Dr. Stillé, resigning his office, in consequence of the impaired state of his health.

On motion of Dr. Arnold, of Savannah, Ga., it was proposed that the letter of Dr. Stillé be placed on record, in compliment to him, for the interest he has manifested in the Association.

Dr. Arnold offered the following resolution, which was adopted:

*Resolved,* That a Committee of one from each State represented in the Association, to be chosen by their respective Delegates, be appointed to nominate suitable officers to be elected for the ensuing year.

On motion of Dr. Frost, the Association took a recess of ten minutes to enable the Delegation to appoint one of their number a member to constitute the Nominating Committee, in compliance with the above resolution.

The President of the Association, at this stage of the proceedings, read an address of some length on matters connected with the profession, and the advancement of medical science, which was well received, and elicited the commendation of those present.

On the reassembling of the Convention, the President reported the following gentlemen, as having been selected as a Committee on Nominations from the different State Delegates, viz.: Dr. Geo. Mendenhall, of Ohio; B. R. Wellford, of Virginia; Jos. Fithian, of New Jersey; R. D. Arnold, of Georgia; G. W. Miltenberger, of Maryland; H. R. Frost, of South Carolina; N. G. Pittman, of North Carolina; W. H. Anderson, of Alabama; A. H. Stevens, of New York; Usher Parsons, of Rhode Island; Joseph Carson, of Pennsylvania; Z. Adams, of Massachusetts; Thos. Reyburn, of Missouri; Jas. Jones, of Louisiana; J. B. Flint, of Kentucky; John Sloan, of Indiana; C. Boyle, of the District of Columbia, and J. B. Lindsley, of Tennessee.

The Nominating Committee, through their Chairman, then read the subjoined names as suitable candidates for officers of the Association for the ensuing year, viz.:

Dr. James Moultrie, of S. C., President.
Dr. Geo. Heyward, of Mass., }
Dr. R. D. Arnold, of Geo., }
Dr. B. R. Wellford, of Va., } V. Presidents.
Dr. J. B. Flint, of Kentucky, }
Dr. H. W. Desaussure, of S. C., } Secretaries.
Dr. P. C. Gooch, }
Dr. Isaac Hays, Treasurer.

On motion of Dr. La Roche, of Pa., the Report was accepted, and the gentlemen thus nominated were elected the officers of the Association for the ensuing year, and were invited to take their seats.

The President elect then took the chair, and in a few appropriate remarks, returned his thanks for the honor thus conferred on him by the Association.

The Secretary read a report transmitted to him from the Committee of Unfinished Business, appointed at the session of 1850.

On motion of Dr. Arnold, of Georgia, the Report was accepted, and laid on the table.

On motion of Dr. Gaillard, of South Carolina, the following resolution, offered by Dr. Drake, of Cincinnati, at the session of 1850, was taken up for consideration.

*Resolved*, That the second section of the Regulations of the Association, be so amended as to require that candidates for membership by invitation, be nominated in writing by five members; that when elected they shall enjoy all the rights of Delegates, and that all permanent members shall be entitled to vote.

After some discussion, on motion of Dr. A. H. Stevens, of New York, the Resolution was referred to a Committee, consisting of Drs. Drake, of Ohio, Wood, of Pa., and Wellford, of Va.

Dr. Stevens, of New York, offered the following Resolution, which was discussed by Drs. Storer of Mass. and Moore, of Ga. and finally rejected.

*Resolved*, That a Committee be appointed to report to the Association the business before it, and to offer such suggestions as they may deem advisable for the due discharge of the same.

On motion, the Association adjourned to meet on Wednesday at 10 o'clock.

*Second day—Morning Session.*

The Convention met pursuant to adjournment, the President in the Chair.

The minutes of the previous meeting were read and confirmed.

Dr. Wood asked and obtained leave to read the following report, on amending the Constitution, on behalf of himself and Dr. B. R. Wellford:

The Committee to whom was referred the Proposition of Dr. Drake, for an alteration of the rules in relation to the admission and rights of members, have the honor to report as follows:

There are two distinct branches of the proposition; the first of which relates to the invitation of medical men, not delegates, to participate in the proceedings of the Association; the second has in view the extension of the right of voting to permanent members.

The Committee agree in the general purport of the first part of the proposition. As it now stands, the rule admits of a too easy admission to the privileges of members, and it is susceptible of great abuse. It might happen, in a place where the number of resident Physicians was very considerable, that sufficient might be introduced to control the decisions of the delegates. To guard against such a result, the Committee recommend, that, in addition to the provision that none should be invited by the Association, unless upon a previous written proposal, by five delegates, the existing rule should be so altered as not to confer upon the invited members the privilege of voting.

In relation to the second part of the proposition, that, namely, which gives the privilege of voting to permanent members, the Committee do not consider its adoption advisable, on the following grounds: This Association is essentially a representative body. Its opinions are supposed to be those of the Societies or Associations by which the delegates are appointed, and go forth to the world with the authority in some degree of the medical profession generally. Now, if permanent members were permitted to vote, they would express their own individual opinions, and support their own individual preferences; both of which might be in direct opposition to those of the delegates, and not fairly representative of general medical sentiment. It is easy to conceive, that combinations among permanent members might be formed, more powerful than the properly delegated body, which might thus be overruled in its decisions. The opinions or wishes of a comparatively few individuals might thus go forth to the world, as those of the profession at large; and private purposes might be answered at the expense of the general good. This would defeat the main objects of the Association, and prevent it from continuing, what it may now be considered to be, the exponent of enlightened medical sentiment in this country.

The Committee, therefore, recommend that the question on Dr. Drake's proposition be taken separately upon its two branches; that the first be adopted with a modification, withholding the right of voting from invited members; and that the second, which confers this right upon permanent members, be not adopted.

Geo. B. Wood,
B. R. Wellford.

*Charleston*, (S. C.,) May 7, 1851.

Dr. Drake then read the subjoined minority report:

The undersigned, a minority of the Committee, to whom was referred the resolution for amending the second section of the Constitution, begs leave to report, that in his opinion, it is expedient and will be found promotive of the great objects for which the Association was formed, that "members by invitation" should not be admitted, except under a written nomination, by five members; that when thus chosen, they should enjoy all the rights and privileges of Delegates, including permanent membership; and that all permanent members should be entitled to vote. With these views the undersigned respectfully submits a revision of the resolution into the following:

*Resolved,* That members by invitation shall be nominated, in writing, by five members, which nomination shall be made a matter of record; that when elected they shall enjoy the rights and privileges of Delegates, and remain as permanent members of the Association.

*Resolved,* That all permanent members shall have the right of voting.

Respectfully submitted. DAN. DRAKE.

Dr. I. Hays moved to take up the majority report, which motion was carried.

Dr. Arnold spoke against the article of the Constitution, authorizing invited members to vote.

Dr. Wood explained his report, and urged its adoption.

Dr. Davis, of Chicago, said that there was much misunderstanding in regard to the intention of the Constitution in respect to the members by invitation. He hoped that the Constitution would be strictly acted up to, and that members should be invited only "from sections not otherwise represented."

Dr. Wood said his was an amendment, and not a repeal of the old provision.

Dr. Drake responded—He had waited for arguments against his resolution, but had heard none. He then entered into a long argument in favor of *popularizing* the Association with the Profession in the United States, and took ground in favor of a permanent place of meeting at Washington City.

Dr. W. Atlee, of Lancaster, said he could see no harm in giving the privilege of voting to invited members who came from unrepresented localities, but was opposed to the right of voting proposed to be given to permanent members.

Dr. Meigs, of Philadelphia, asked whether a gentleman would be invited to attend without any privileges, and went on to say that he hoped the Convention would have five, ten or even twenty thousand in attendance at some future period.

Dr. Hooker, of Connecticut, begged to be allowed to offer the following Resolution, the Resolution of Dr. Drake having been laid on the table for the present.

*Resolved,* That no member be permitted to speak longer than 10 minutes at one time in any one debate.

Dr. Phelps, of New York, offered to amend the Resolution by inserting "15," which motion was lost.

The Resolution as offered by Dr. Hooker was then adopted.

Dr. Hays moved to lay the subject on the table, and added that by a constitutional provision it was required to lay over one year.

The motion was seconded by Dr. Tucker, of Virginia.

Dr. Dickson, of South Carolina, asked if the motion swept off the whole Resolution, and was answered affirmatively by Dr. Hays.

Dr. ——— said if the matter was postponed now, they would not be out of difficulty, because all that is necessary to defeat it next year would be to move to amend it, and it would have to lay over a year again, &c.

The matter was finally laid on the table.

Dr. Wood, of Pennsylvania, called up the second part, or that portion giving to permanent members the right to vote.

The majority committee accepted the substitute of the minority committee, which was read as follows, viz.:

*Resolved,* That all permanent members shall have the right of voting.

Dr. Dickson urged the adoption of the above Resolution.

Dr. Hays, of Philadelphia, remarked that the Constitution had not been studied by the gentleman who had urged the adoption of the Resolution; and spoke in opposition to the measure.

Dr. Thomson, of Delaware, supported Dr. Hays, and hoped that the whole matter would be laid over for a year.

Dr. Dickson observed that he had been accused of ignorance of the Constitution. He hoped to have these gentlemen always here to instruct him.

Dr. Bond, of Maryland, took part in the discussion.

Dr. Adams, of Massachusetts, remarked that they ought to strike out the words—"Permanent Delegates"—from the Constitution, and was proceeding with his remarks, when the gentleman was called to order.

The question was here taken on the adoption of the Resolution, which was lost by a large majority.

Dr. I. Hays, the Treasurer of the Association, then read the Report of the Committee of Publication, and also the Report of the Treasurer.

The subjoined resolutions, appended, were read and adopted.

1. *Resolved,* That the assessment for the present year shall be three dollars.

2. *Resolved,* That those Delegates who pay the assessments, shall be entitled to one copy of the transactions for the present year; and that the payment of two dollars, in addition, shall entitle them to two additional copies.

3. *Resolved,* That permanent members shall be entitled to one copy of the transactions for the present year on the payment of two dollars, and three copies on the payment of five dollars.

4. *Resolved,* That Societies which have been represented in the Association shall be entitled to copies for their members on the same terms that copies are furnished to permanent members.

5. *Resolved,* That permanent members, unless present at the meeting as Delegates, shall not be subject to any assessment.

6. *Resolved,* That any Delegate who is in arrears for his annual assessment shall not be considered as a permanent member.

7. *Resolved,* That the several Committees be requested to bring to the meeting of the Association, their reports, correctly and legibly transcribed; and that they be required to hand them to the Secretaries as soon as they have been read.

All which is respectfully submitted. Isaac Hays,
*Philadelphia,* April 20, 1851. D. Francis Condie.

Dr. Drake, of Ohio, moved that the Report on Surgery be read first. Adopted.

Professor Eve, Chairman of Committee on Surgery, then proceeded to read his report.

A motion was made by Dr. Davis to commit the same to the Committee on Publication, which was adopted.

Dr. Hays moved to read Dr. Flint's Report by its title—Practical Medicine—and refer the same to the Committee on Publication, which motion was adopted, and several hundred copies printed and furnished by the author were directed to be distributed.

A motion was then made to adjourn till 5 o'clock, P. M., which was adopted, and the Convention adjourned.

*Afternoon Session.*

The President having organized the meeting,

Dr. Boyle, of Washington, offered a resolution that the Association in future meet at Washington City.

Dr. C. P. Johnson, of the Virginia Medical Society, extended an invitation to meet at Richmond; Dr. Jones, of the University of Louisiana, to meet in New Orleans; Dr. J. P. Johnson, of Missouri, to meet in St. Louis. The resolution and invitations were referred to the Committee on Nominations.

The President suggested to the Society the propriety of appointing the Standing Committees at an early date.

Dr. Wood remarked that there was a proposition to abolish Standing Committees.

Dr. Hays said he was opposed to these Committees, but would not press an alteration.

Dr. Tucker moved that the appointment of the Standing Committees be referred to the Committee on Nominations, which motion was adopted.

Dr. Jones, of Louisiana, resigned as a member of the Committee of Nominations; and Dr. Fenner, of New Orleans, was appointed in his place.

Dr. Parsons then moved that the Committee on Nominations be requested to resume its labors, which was adopted.

Dr. Wragg, of Charleston, moved that the Report of the Committee on Prize Essays be read, and then that the Obstetric Report be brought up.

The Report on Prize Essays was then read, and certain resolutions appended thereto were adopted.

When, on motion of Dr. Ready, of South Carolina, the whole matter was referred to the Committee of Publication.

Dr. Storer, of Boston, Chairman of the Committee on Obstetrics, read the Report on that subject. He stated that he had received a letter from Dr. Thompson, of Illinois—that he was the only member of the Committee who had aided him in any degree. He mentioned this fact, because he had to hold himself entirely responsible for all the inaccuracies, &c.

Dr. Phelps, of New York, moved that the Report be referred to the Committee of Publication.

Dr. Robertson, of South Carolina, moved that the statistics, alluded to in the Report, be stricken out, as the author of them was not a reliable man.

Dr. Storer seconded the motion.

Dr. Bond moved to postpone the Report until morning, which was seconded by Dr. Gilman.

A short debate here ensued; when it was finally agreed to re-commit said portion of the Report, to be corrected and laid before the Association in the morning.

On motion, the Convention adjourned.

*Third day, Morning Session.*

The President, Dr. James Moultrie in the Chair.

The Minutes of the previous meeting were read, and after some slight amendments were confirmed.

Dr. J. M. Smith, of Mass., moved that the Report of the Committee on Medical Education be made the special order, after the disposal of the Report of the Committee on Obstetrics.

Dr. Gaillard, on behalf of the Committee of Arrangements, read a list of Delegates as registered since the last report.

Dr. Campbell, of Georgia, presented a model of a mal-formation of the knee joint, the patella being absent.

Dr. Wood, of Pennsylvania, offered the following resolution:

*Resolved,* That Colleges, exclusively of Dentistry and Pharmacy, are not recognized by the Association, as among the bodies authorized to send Delegates to its meetings.

Dr. Wood, of New York, moved to amend, by dividing the Resolution, so as to take the question, first, on the reception of Delegates from Colleges of Dentistry; secondly, on the reception of Delegates from Colleges of Pharmacy.

The amendment having been accepted, the question of the reception of Delegates from Colleges of Dentistry was debated.

Dr. Lamb moved an indefinite postponement of the Resolution, which was lost.

Dr. Yardley, of Pennsylvania, asked and obtained leave to read the subjoined Resolution, presented by the Philadelphia County Medical Society.

*Resolved,* That all the Medical Colleges in the United States are hereby earnestly and respectfully requested to hold a Convention, through Delegates respectively chosen by them at least once in every six years, to take into consideration the proper method of harmoniously elevating the standard of Medical education in the said Colleges.

The discussion of the original question was then resumed.

A motion was finally made by Dr. Hays, of Pennsylvania, that the whole resolution of Dr. Wood, including colleges of Dentistry and Pharmacy, be referred to a special committee of five members, which resolution was adopted.

On motion of Dr. Yardley, of Pennsylvania, the resolution presented by the Philadelphia County Medical Society was also sent to the same Committee.

Dr. Jones, of North Carolina, offered the following resolution:

*Resolved,* That all the Medical Colleges in the United States, are hereby earnestly and respectfully requested to hold a Convention, through Delegates respectively chosen by them, at least once in every six years, to take into consideration, the proper method of harmoniously elevating the standing of Medical Education in the said Colleges.

The order of the day was then called up, when Dr. Storer reported that he had erased the statistics referred to yesterday, and that he placed the report in the hands of the Association. Dr. S. said that there was objection to the remarks on the subject of Dr. Gilman's paper on the speculum uteri. He asked that he be permitted to remove the unnecessary expression of opinion in regard to that subject. He further added that he had taken from the journals these facts, &c., and was not therefore responsible for the correctness of the papers, &c.

Dr. Bond, of Md., remarked that there were charges in these reports which he did not individually endorse; but which got out in a book under the sanction of the Association.

On motion of Dr. Davis, the report was referred to the Committee on Publication.

At this stage of the proceedings, Professor S. S. Haldeman, of Lancaster City, Pennsylvania, through Dr. John L. Atlee, presented to the Association an Essay on Latin Pronunciation, of which he is the author; and which, on motion of Dr. Atlee, was referred to the Committee on Medical Literature.

On motion, the regular order was suspended for the reception of the Report of the Committee of Nominations which was read and laid on the table.

Dr. Hays then called up the Resolution on page 43, vol. 2, of the transactions of the Association, and moved to strike out "all that relates to Committees," &c.

The motion was seconded by Dr. Stevens, and urged by Dr. Drake, who read some ten or twelve special points, which he said ought to occupy the Association instead of being occupied with Epitomes of Rankin & Braithwaite.

Dr. Hooker, of Conn., spoke of the looseness of Committees and editors of the Journals.

Dr. Davis thought that they could decide on the matter at once.

Dr. Hays proposed to dispense with the Standing Committees. The question was then taken on the resolution, which was adopted.

Dr. Wood, of Pennsylvania, offered the following resolution, which was adopted:

*Resolved,* That a Committee be appointed to take into consideration the arrangement of a Committee for future action, to report as speedily as possible.

The Chairman of the Committee on Medical Education was about to read the regular report on that subject, when Dr. Drake moved the suspension of the reading till after the recess, as it was a very long report.

On motion of Dr. Johnston, of Missouri, the report of the Committee on Medical Literature was then taken up.

Dr. Desaussure announced that Dr. Davis would read a paper entitled an experimental enquiry concerning some points connected with the process of Assimilation and Nutrition.

Dr. Reyburn, of Missouri, presented and read the Report of the Committee on Medical Literature. In the Course of his reading the Report, he gave way to a motion to adjourn.

*Afternoon Session.*

The President took the Chair at half past 5 o'clock and organized the meeting.

The Secretary announced the following gentlemen as having been appointed by the President, under Resolution of this morning, concerning a Committee for the arrangement of business, for the occupation of the Association in future: Drs. G. B. Wood, of Pennsylvania; I. Hays, D. Drake, A. H. Stevens, W. Hooker, B. R. Wellford, and S. H. Dickson.

The following gentlemen were appointed a Committee under a resolution in regard to Schools of Pharmacy and Dental Surgery, viz: Drs. Hays, Stevens, Yardley, Storer and Jones.

Dr. Dickson moved the following Preamble and Resolutions, which were seconded by Dr. Bond, and unanimously adopted without debate.

Whereas, efforts are being made to repeal the law of 1847, which confers protective rank on the members of the Medical Department of the Army, therefore

*Resolved*, That the American Medical Association, views with regret the existence of hostility to the act of Congress, approved February 11, 1847, which confers legal rights, and equality with other Staff Departments, on the Medical officers of the Army, and gives them a position to which the importance and character of the profession entitles them.

*Resolved*, That copies of these resolutions, with the resolutions of the Association, passed at its last annual meeting, on the same subject, be transmitted to the Secretaries of War and of the Navy, through the Chiefs of the Medical Department of each service, and to the presiding officers of the Senate and House of Representatives of the United States.

The reading of the Report of the Committee of Medical Literature was then concluded.

On motion, the Report was adopted, and referred to the Committee on Publication.

The Report of the Committee on Medical Education was then called for, and as the hour was late, the Chairman read only so much of it as relates to Demonstrative Midwifery, which had by special resolution been referred to the Committee.

On motion, the Report was accepted, and referred to the Committee of Publication.

Dr. Dickson presented the following Resolution, which was adopted:

*Resolved*, That this Association unanimously approve of the opinions expressed in the Report of the Committee on Medical Education in respect to Demonstrative Midwifery.

The Convention then adjourned.

*Fourth day, Morning Session.*

The President, Dr. James Moultrie, in the chair.

The minutes of the last meeting were read and confirmed.

The report of the Committee on Medical Education being the special order, Dr. Stevens, of New York, asked and obtained leave to introduce the following resolutions;

*Resolved,* That the members of this Association cannot separate without expressing their grateful sense of the hospitalities, and numerous delicate attentions received from their Medical brethren of South Carolina, and the citizens of Charleston.

*Resolved,* That a Committee be formed to procure a tablet with a suitable inscription, commemorative of this meeting, and the feeling it has elicited ; to be placed at the disposal of the Medical Association of South Carolina.

This tablet is here placed by the American Medical Association, to commemorate their annual meeting in the city of Charleston, in May, 1851, and to signalize their gratitude for the extraordinary professional and social enjoyments that accompanied it.

The resolutions having been seconded were adopted; and Dr. Stevens, further moved that Drs. Hayward, of Mass. and F. A. Ramsey, of Tenn. and himself constitute the Committee.

Dr. Ramsey, of Tenn., asked and obtained leave to read a letter from Dr. E. D. Fenner, of Louisiana, and offered the following resolution on the subject, which was adopted.

*Resolved,* That the efforts of Dr. Fenner, to place on a firm and durable basis, an annual publication, embracing Medical Reports from the whole Southern portion of the Union, merits the commendation of this Association, and should receive solid support from American Physicians.

Dr. Hays, of Pa. asked and obtained leave to call up for consideration, so much of the report of the Nominating Committee, as relates to the selection of the next place of Meeting of the Association, and the appointment of the Committee of Arrangements and the Committee of Publication, the other Standing Committees having been abolished. The report having been read, Dr. Drake, of Ohio, made an urgent appeal in favor of Washington City as the next place of meeting. The question being taken on the adoption of that part of the Report of the Committee, which proposed Richmond, (Va.) as the next place of Meeting, it was adopted by a large majority. The question being taken on the confirmation of the Committee of Arrangements and Publication, the nominations of the Committee were confirmed.

Richmond, Va., was selected as the next place of Meeting by the Association, and the following gentleman appointed a Committee of Arrangements, viz :—Drs. R. W. Haxall, Chairman ; Carter P. Johnson, James Beale; Chas. B. Gibson, S. Maupin, R. D. Haskins, C. S. Mills and M. P. Scott. Committee of Publication—Drs. Hays, of Pa., G. Emerson, of Pa., D. F. Condie, of Penn., H. W. Desaussure, of So. Ca., I. Parrish, of Penn., P. C. Gooch, of Va., and G. W. Norris, of Penn.

Dr. Hooker, of Conn., Chairman of the Committee on Medical Education, completed the reading of the report of the committee, and offered the following resolutions:

*Resolved,* That the abuses which exist in the modes of Medical Education pursued in this country, demand the serious consideration of the profession.

*Resolved,* That free discussion in relation to the causes is an important means of effecting their removal.

*Resolved,* That in the opinion of this Association no effort to remove these abuses can succeed, that is not based upon a reform in the public sentiment, both of the profession and of the community.

*Resolved,* That this reform, so far as the profession is concerned, is to be effected mainly through its organization, and that it is therefore incumbent upon every Physician to do all that he can to give them character and efficiency.

*Resolved,* That this Association have confidence in all proper efforts which have for their object a reform in the sentiments and practice of of the community in relation to Medicine and the Medical Profession.

*Resolved,* That the recommendations of this Association at its former meetings in regard to Education, both preliminary and medical, be reaffirmed, and that both the schools and private preceptors be still urged so to do their duty as to secure to the community a well educated profession.

*Resolved,* That in the work of medical reform, while all precipitate movements should be avoided, we should aim at a steady advance, from year to year, till a thorough system of education be established by the profession throughout our country.

Dr. Wood, of Pennsylvania, asked leave to suspend the order usually taken with Reports. Permission being granted, he read the following report, which was adopted:

The Committee to whom was referred the subject of arranging a plan of Committees, for future action, in place of the Standing Committees abolished by the Association, have the honor to report, as follows:

It appears to them that the most feasible plan of accomplishing the objects of the Association, is to select certain subjects, which may be considered as suitable for investigation, and to refer these subjects to special committees, to be appointed before the close of the present session, and to report to the next. Such a selection the Committee have accordingly made, and will offer to the consideration of the Association.

As an additional means of securing valuable contributions, they propose, also, the appointment of a committee, whose business it shall be, in the interval between this and the next session, to receive original volunteer papers, upon any subject which their authors may choose; to decide upon the merits of these papers; and to present to the Association, at its next session, such of them as they may deem worthy of receiving this direction. With a view to increase competition, they think it advisable that a prize of fifty dollars, or a gold medal of that value, be awarded to each of the five papers presented to the Association, or any smaller number of them, which the committee may consider most meritorious, and the Association may resolve to publish.

In reference to the resolution presented in the report of the Standing Committee on Medical Literature, and referred to the present Committee, they have only to observe that, as its ends will probably be most effectively obtained, by the adoption of the general plan which they have already brought before the notice of the Association, they do not consider it expedient to make any further report.

As to the appointment of the Special Committees referred to, your committee think that the most convenient plan will be to refer to a Special Committee, the nomination of a Chairman for each, who shall then select, at his convenience, two individuals, to aid him, with the restriction only, that the persons so selected, shall be members of the Association.

To the same Nominating Committee, may be referred the appointment of the General Committee, whose business will be to receive and judge, whatever papers, may be submitted to them. As the members of this General Committee must frequently compare opinions it will be desirable that they should reside near each other; and it is accordingly proposed, that they should be chosen from one neighborhood. If the plan be found to work well, this locality may be changed every year, so that each section of the Union may, in its turn, be charged with this duty. The committee would suggest that the General Committee should be first chosen from members of the Association residing in Boston or its neighborhood, as the most northern point.

To embody these suggestions in due form, the Committee offer the following resolutions:

I. *Resolved*, That Committees of Three be appointed to investigate and report severally on the following subjects:

1st. Causes of the Tubercular diathesis.

2d. Blending and conversion of the types of fever.

3d. The mutual relations of Yellow Fever and Bilious Remittent Fever.

4th. Epidemic Erysipelas.

5th. Acute and Chronic diseases of the Neck of the Uterus.

6th. Dengue.

7th. The Milk Sickness, so called.

8th. Endemic prevalence of Tetanus.

9th. Diseases of Parasitic origin.

10th. Physiological peculiarities and diseases of negroes.

11th. The action of water on lead pipes and the diseases which proceed from it.

12th. The alkaloids which may be substituted for quinia.

13th. Permanent cure of reducible hernia.

14th. Results of surgical operations for the relief of malignant diseases.

15th. Statistics of operations for removal of stone in the bladder.

16th. Cold water dressings.

17th. The sanitary principles applicable to the construction of dwellings.

18th. The toxicological and medicinal properties of our cryptogamic plants.

19th. Agency of the refrigeration produced through upward radiation of heat as an exciting cause of disease.

20th. Epidemic diseases of New-England and New-York.

21st. Epidemic diseases of Pennsylvania, New Jersey, Delaware and Maryland.

22d. Epidemic diseases of Virginia and North-Carolina.

23d. Epidemic diseases of South-Carolina, Georgia, Florida and Alabama.

24th. Epidemic diseases of Mississippi, Louisiana, Texas and Arkansas.

25th. Epidemic diseases of Tennessee and Kentucky.

26th. Epidemic diseases of Missouri, Illinois, Iowa, and Wisconsin.

27th. Epidemic diseases of Indiana, Ohio and Michigan.

II. *Resolved*, That a Committee of Nomination be appointed, whose duty it shall be to nominate one Chairman for each of the above Committees.

III. *Resolved*, That each of the Chairmen thus nominated shall select, at his earliest convenience, the members of the Association to complete the Committee.

IV. *Resolved*, That a Committee of five members be appointed, to be called the Committee for Volunteer Communications, whose duty it shall be, in the interval between the present and the next succeeding sessions, to receive papers upon any subject from any persons who may choose to send them, to decide upon the merits of these papers, and to select for presentation to the Association, at its next session, such as they may deem worthy of being thus presented.

V. *Resolved*, That the Committee for Volunteer Communications, shall have the power to form such regulations as to the mode in which the papers are to be presented, and as to the observing of secrecy, or otherwise, as they may think proper.

VI. *Resolved*, That the selection of the members of this Committee be referred to the same Nominating Committee, whose duty it will be to appoint the Chairman of the several Special Committees, as above directed; with this restriction, that the individuals composing it shall reside in the same neigborhood.

VII. *Resolved*, That a prize of fifty dollars be awarded to each of the volunteer communications reported on favorably by the Committee, and directed by the Association to be published: Provided, that the number to which the prize is thus awarded do not exceed five; and provided, also, if the number approved and directed to be published exceed five, that in such case, the prize shall be awarded to the five which the Committee may determine to be the most meritorious. All of which is respectfully submitted. Geo. B. Wood, *Chairman.*

*Charleston*, May 9th, 1851.

Dr. Hays, of Penn., gave notice, that at the next meeting of the Association, he should offer an amendment to the Constitution, line 4, so as to read $10 instead of $3.

Dr. Atlee, of Pennsylvania, remarked on the value of the Report of the Committee on Medical Education, and offered the following resolution, which was adopted:

*Resolved,* That it be recommended to the several State Medical Societies throughout the Union, to procure a re-publication of the Report of the Committee on Medical Education, for general distribution among the profession.

Dr. Drake offered the following resolution:

*Resolved,* That in the opinion of the Association, the students of our schools should be required to matriculate within the first days after the opening of the Sessions, and continue their attendance to the end of the terms, taking with them evidence of the same, to be presented with tickets of the Professors when they become candidates for Degrees.

The resolution was adopted, and Dr. Gibson moved to defer the filling up of the blank. Some discussion arose on this point, when the resolution was left to read, "within the first days," &c.

The report of the Committee on Medical Science was then called up, when a letter was read from Dr. Dowler, Chairman of said Committee, regretting his inability to be present, and the necessity of sending it.

Dr. Fenner then read the outlines of the report, and asked permission to retain the same for revision, copying, &c. which was granted.

Dr. Mauran offered the following resolution, which was adopted.

*Resolved,*That the Committee on Publication, be instructed to print conspicuously upon the title page of the forthcoming volume of the transactions, the following declaration, viz: "The American Medical Association, although formally accepting and publishing the Reports of the various Standing Committees, holds itself wholly irresponsible for the opinions, theories or criticisms, therein contained.

Dr. Storer moved the following Resolution, which was adopted:

*Resolved,* That the hearty thanks of this Association be presented to their late Secretary, Alfred Stillé, M. D. for his constant, unwearied and invaluable services since its first organization.

The report of the Committee on Adulterated Drugs was read. A motion was made to refer the same to the Committee on Publication, which was lost, and a motion to lay it on the table, adopted.

Dr. Gaillard, of South Carolina, Chairman of the Committee on Hygiene, presented an outline of the Report on that subject. Referred to the Committee of Publication, with authority to append thereto a paper now in preparation, on the Mortuary Statistics of certain cities.

Dr. Drake, of Ohio, offered the following amendments to the Constitution, which were read and ordered to lie over under the rule:

All members by invitation, must be nominated in writing by five members of the Association, whose names shall be recorded in the minutes. When elected, they shall enjoy all the rights and privileges of Delegates, and remain permanent members of the Association.

All permanent members shall be entitled to vote, and when they attend a meeting of the Association, their respective names shall be registered, and each shall pay the sum required from a delegate.

The Secretary read a Protest from the Iowa University, against the representation of Rush Medical College in this Association.

Dr. Jervey moved to refer the Protest to a Special Committee, to report at once.

Dr. Wood moved to refer it to the Committee on Colleges of Pharmacy and Dentistry, which was carried. Dr. Jervey withdrawing his motion.

Dr. Wood read the following Report of the Committee of Nominations, which was adopted:

The Committee to whom was referred the nomination of the Chairman of the several Special Committees, to report at the next session, and also, of the committee for volunteer communications, report that they have fulfilled the object of their appointment, and offer the following list of Chairmen, to the committees first referred to, viz:

1st. Dr. F. Condie, Philadelphia, Chairman of the Committee on the causes of the Tubercular Diathesis.

2d. Dr. S. H. Dickson, of Charleston, S. C., on the blending and conversion of the Types of Fever.

3d. Dr. James Jones, of New-Orleans, on the mutual relations of Yellow and Bilious Remittent Fevers.

4th. Dr. John B. Johnson, of St. Louis, Mo., on Epidemic Erysipelas.

5th. Dr. Charles D. Meigs, of Philadelphia, Acute and Chronic diseases of the Neck of the Uterus.

6th. Dr. J. P. Jervey, of Charleston, S. C., on Dengue.

7th. Dr. Daniel Drake, of Cincinnati, Milk Sickness—so called.

8th. Dr. Lopez, of Mobile, Alabama, Epidemic prevalence of Tetanus.

9th. Dr. Geo. B. Wood, of Philadelphia, on diseases of Parasitic Origin.

10th. Dr. R. D. Arnold, of Savannah, Geo., on the Physiological Peculiarities, and diseases of Negroes.

11th. Dr. Horatio Adams, of Waltham, Mass., on the Action of Water on lead pipes, and the diseases which proceed from it.

12th. Dr. Jos. Carson, of Philadelphia, on the Alkaloids which may be substituted for quinia.

13th. Dr. Geo. Hayward, Boston, Massachusetts, on the Permanent Cure of Reducible Hernia.

14th. Dr. S. D. Gross, Louisville, Kentucky, on Results of Surgical Operations for the relief of Malignant Diseases.

15th. Dr. James R. Wood, New-York, Statistics of the Operation for the Removal of Stone in the Bladder.

16th. Dr. Charles A. Pope, St. Louis, Missouri, Water, its topical uses in Surgery.

17th. Dr. Alex. A. Stevens, New-York, Sanitary principles applicable to the Construction of Dwellings.

18th. Dr. Porcher, Charleston, South-Carolina, Toxicological and Medicinal Properties of our Cryptogamic Plants.

19th. Dr. G. Emerson, Philadelphia, Agency of the Refrigeration produced through upward Radiation of Heat, as an exciting cause of disease.

20th. Dr. Worthington Hooker, Connecticut, on the Epidemics of New-England and New-York.

21st. Dr. John L. Atlee, of Lancaster, Penn., on the Epidemics of New Jersey, Pennsylvania, Delaware and Maryland.

22d. Dr. Robert W. Haxall, Richmond, Va., on the Epidemics of Virginia and North Carolina.

23d. Dr. Wm. M. Boling, Montgomery, Ala., on the Epidemics of South Carolina, Georgia, Florida and Alabama.

24th. Dr. Ed. H. Barton, Louisiana, on the Epidemics of Mississippi, Louisiana, Texas and Arkansas.

25th. Dr. Sutton, Georgetown, Ky., on the Epidemics of Tennessee and Kentucky.

26th. Dr. Thos. Reyburn, Missouri, on the Epidemics of Missouri, Illinois, Iowa and Wisconsin.

27th. Dr. Geo. Medenhall, Ohio, on the Epidemics of Ohio, Indiana and Michigan.

The following gentlemen were appointed on the Committee for Volunteer Communications, viz:—Drs. Geo. Hayward, S. B. J. Jackson, D. H. Storer, and Jacob Bigelow, of Boston, and Dr. Usher Parsons, of Providence, R. I.

Signed on behalf of the Committee,

Geo. B. Wood, *Chairman.*

*Charleston*, Friday, May 9th. 1851.

The President read an invitation from the Committee of Reception, to a steamboat Excursion.

Dr. M'Intyre of New-York, proposed that the code of Ethics and Constitution of the Association be recommended to published by the several State Societies. Proposition adopted.

Dr. Grimshaw offered the following resolution:

*Resolved*, That Medical Colleges, in publishing Statements of the number of Medical and Surgical cases treated at their Dispensaries, act contrary to the spirit of the Code of Ethics adopted by this body.

Adjourned.

*Afternoon Session.*

The Association re-assembled at 5 o'clock, Dr. B. R. Wellford of Virginia, Vice President, in the chair.

The special order was called for, and Dr. Davis, of Ill., read a paper on the influence of certain diet on the functions of Respiration and Calorification, &c.

The President, Dr. James Moultrie, resumed the chair.

Dr. Hays moved to proceed with the consideration of unfinished business.

Dr. Grimshaw offered the subjoined Resolution, which was adopted.

*Resolved*, That the thanks of the Association be returned to Dr. Davis for the paper just presented by him.

Dr. F. A. Ramsey, of Tenn., called up as unfinished business, the Resolution offered yesterday by Dr. Jones, of Tenn., and not then acted upon, to which Dr. Grimshaw offered the following Amendment—"And that the first Convention be held before the first of May 1852."—The question being taken on the Resolution and the Amendment, they were both negatived by a large majority.

Dr. Phelps, of New York, offered the following Resolutions, which were unanimously adopted:

*Resolved*, That the warmest thanks of the Association be tendered to the Trustees of the St. Andrew's Society, for the gratuitous use of their very convenient and eligible Hall; and to all those other Institutions and Reading Rooms, which have been so freely thrown open for the inspection and use of the members.

*Resolved*, That the Committee of Arrangements receive our most grateful acknowledgments for the very handsome and indeed magnificent manner in which they have provided for the entertainment and pleasure of the Delegates from abroad, during their sojourn in the City of Carleston.

*Resolved*, That not only the Profession of Medicine, but also private munificence, and the kind attentions of the citizens generally, have conspired in manifestation of that urbanity of manner, and that unwearied and kind attention, which commands not only our profound admiration, but will be followed by the most pleasing recollections so long as life and thought shall endure.

On motion of Dr. Stevens, the above resolutions, with those offered by him at the Morning Session, were ordered to be published in the city papers.

Dr. Johnson, of St. Louis, moved to adjourn *sine die*, which was adopted.

The Vice President, Dr. Wellford of Virginia, then congratulated the Association on the happy termination of its labors, and declared it adjourned to meet again in Richmond, Va., on the first Tuesday in May next.

[For a large portion of the above report we are indebted to the courtesy of the Secretary, Dr. Gooch, who will please accept our thanks.—Eds. Exam.]

